# A class of stochastic Gronwall’s inequality and its application

**DOI:** 10.1186/s13660-018-1932-3

**Published:** 2018-12-10

**Authors:** Xin Wang, Shengjun Fan

**Affiliations:** School of Mathematics, China University of Mining and Technology, Xuzhou, China

**Keywords:** Gronwall’s inequality, Stochastic, Backward stochastic differential equation, Comparison theorem

## Abstract

This paper puts forward the basic form of stochastic Gronwall’s inequality and uses, respectively, the iterative method, the integral method and the martingale representation method to prove it. Then it presents an application to prove a comparison theorem of $L^{p}$ solutions for one-dimensional backward stochastic differential equations under the stochastic Lipschitz condition.

## Introduction

Gronwall’s inequality was first proposed and proved as its differential form by the Swedish mathematician called Thomas Hacon Gronwall [1] in 1911. The integral form was proved by the American mathematician Bellmen [2] in 1943; see the following Proposition 1. Gronwall’s inequality is an important tool to obtain various estimates in the theory of ordinary and stochastic differential equation. In particular, it provides a comparison theorem that can be used to prove uniqueness of a solution to the initial value problem.

### Proposition 1

*Assume that* $a\geq 0$ *and* $0< T\leq +\infty $. *Let* $\beta (t)$ *and* $\mu (t)$ *be two non*-*negative continuous functions defined on* $[0,T]$, *which satisfy*

$$ \mu (t)\leq a + \int _{0}^{t}\beta (s)\mu (s)\,\mathrm{d}s, \quad t \in [0,T]. $$

*Then*

$$ \mu (t)\leq a e^{\int _{0}^{t}\beta (s)\,\mathrm{d}s}, \quad t\in [0,T]. $$

With the development of differential equations, it was found that the original Gronwall inequality can no longer meet the needs of application, so people began to study the generalized Gronwall inequality. LaSalle [3] and Bihari [4] have put forward a nonlinear generalization of the Gronwall–Bellman inequality called the Bihari–LaSalle inequality. Pachpatte [5] has presented some other variants and generalizations of Gronwall’s inequality. In recent years, Chen et al. [6] have established some new nonlinear Gronwall–Bellman–Ou–Iang type integral inequalities with two variables, which can be used as a simple tool to study the qualitative and quantitative properties of solutions to differential equations. Lin [7] has provided several generalizations of Gronwall’s inequality and presented their applications to proving the uniqueness of solutions for fractional differential equations with various derivatives. Zhang et al. [8] have studied a class of more general discrete fractional order Gronwall’s inequality by using the definition of a new fractional order integral. Xu et al. [9] have developed some generalized discrete fractional Gronwall inequalities based on an iteration method, which can be used in the qualitative analysis of the solutions to fractional difference equation and summation equation. In particular, Fan et al. [10] have proposed the following backward Gronwall inequality, and used it to study the existence and uniqueness of solutions to backward stochastic differential equations.

### Proposition 2

*Assume that* $a\geq 0$, $0< T\leq +\infty $ *and* $\beta (t):[0,T]\mapsto [0,+\infty )$ *satisfies* $\int _{0}^{T}\beta (s)\,\mathrm{d}s<+\infty $. *Let* $\mu (t):[0,T]\mapsto [0,+\infty )$ *be a continuous function with* $\sup_{t\in [0, T]}\mu (t)<+\infty $ *and satisfy*

$$ \mu (t)\leq a + \int _{t}^{T}\beta (s)\mu (s)\,\mathrm{d}s, \quad t \in [0,T]. $$

*Then*

$$ \mu (t)\leq ae^{\int _{t}^{T}\beta (s)\,\mathrm{d}s}, \quad t\in [0,T]. $$

This paper will put forward a class of stochastic Gronwall’s inequalities, provide its three proof methods based on Propositions 1 and 2, and it will introduce an application in the field of backward stochastic differential equations.

## Notation

In this paper, let $(\varOmega ,\mathcal {F},\mathbf{P})$ be a complete probability space carrying a standard *d*-dimensional Brownian motion $(B_{t})_{t \ge 0}$ and $(\mathcal {F}_{t})_{t\ge 0}$ be the completed natural *σ*-algebra generated by $(B_{t})_{t\ge 0}$. We always assume that $0< T\leq +\infty $, $\mathcal {F}_{T}:=\mathcal {F}$ and $(\mathcal {F}_{t})_{t\ge 0}$ is right-continuous and complete. $\mathbf {E}[\xi ]$ is used to express the mathematical expectation of the random variable *ξ*, $\mathbf {E}[\xi |\mathcal {F}_{t}]$ represents the conditional mathematical expectation of the random variable *ξ* with respect to $\mathcal {F}_{t}$, and $\|\xi \|_{\infty }$ represents the infinity norm of the essentially bounded random variable *ξ*, i.e.,

$$ \Vert \xi \Vert _{\infty }:=\sup \bigl\{ x:\mathbf{P}\bigl( \vert \xi \vert >x\bigr)>0\bigr\} . $$

Denote $a^{+}:=\max \{a,0\}$, $\mathbf {R}:=(-\infty ,+\infty )$, $\mathbf {R}_{+}:=[0, +\infty )$. Let $\mathbf{1}_{A}$ represent the indicator function of the set *A*, which means for each subset $A\subset \varOmega \times [0,T]$, $\mathbf{1}_{A}(\omega ,t) =1$ in the case of $(\omega ,t)\in A$, otherwise $\mathbf{1}_{A}(\omega ,t)=0$.

For each $p>0$, let $L^{p}(\varOmega , \mathcal {F}_{T}, \mathbf{P})$ be the set of all **R**-valued, $\mathcal {F}_{T}$-measurable random variables *ξ* such that $\mathbf {E}[|\xi |^{p} ]<+\infty $. And let $\mathcal {S}^{p}(0,T;\mathbf {R})$ (or $\mathcal {S}^{p}$ simply) denote the set of **R**-valued, $(\mathcal {F}_{t})$-adapted and continuous processes $(Y_{t})_{t \in [0,T]}$ such that

$$ \Vert Y \Vert _{\mathcal {S}^{p}}:= \Bigl(\mathbf {E}\Bigl[\sup_{t\in [0,T]} \vert Y_{t} \vert ^{p} \Bigr] \Bigr) ^{{\frac{1}{p}}\wedge 1}< + \infty . $$

Finally, let $M^{p}(0,T;\mathbf {R}^{d})$ (or $M^{p}$ simply) represent the set of $(\mathcal {F}_{t})$-progressively measurable $\mathbf {R}^{d}$-valued processes $(Z_{t})_{t\in [0,T]}$ such that

$$ \Vert Z \Vert _{M^{p}}:= \biggl(\mathbf {E}\biggl[ \biggl( \int _{0}^{T} \vert Z_{t} \vert ^{2}{\,\mathrm{d}}t \biggr) ^{\frac{p}{2}} \biggr] \biggr)^{\frac{1}{p}\wedge 1}< +\infty . $$

## Main result

We first give the basic form of a stochastic Gronwall’s inequality, which is the main result of this section.

### Theorem 1

*Let* $a>0$ *and the* $(\mathcal {F}_{t}) _{t\geq 0}$-*progressively measurable random process* $\beta (\omega , t): \varOmega \times [0, T]\mapsto \mathbf {R}_{+}$ *satisfy*

$$ \biggl\Vert \int _{0}^{T}\beta (\omega ,s)\,\mathrm{d}s \biggr\Vert _{\infty }< + \infty , $$

*i*.*e*., *there exists a constant* $M>0$ *such that* $\int _{0}^{T} \beta ( \omega , s) \,\mathrm{d}s\leq M$, $\mathrm{d}{\mathbf{P}}\textit{-a.s.}$ *If the* $(\mathcal {F}_{t}) _{t\geq 0}$-*progressively measurable random process* $\mu (\omega ,t):\varOmega \times [0,T]\mapsto \mathbf {R}_{+}$ *satisfies*

$$ \mathbf {E}\Bigl[\sup_{t\in [0,T]}\mu (\omega ,t) \Bigr]< +\infty $$

*and*

1 $$\begin{aligned} \mu (t)\leq a + \mathbf {E}\biggl[ \int _{t}^{T}\beta (s)\mu (s)\,\mathrm{d}s \Big|\mathcal {F}_{t} \biggr], \quad t\in [0,T], \end{aligned}$$

*then*, *for each* $t\in [0,T]$, *we have*

2 $$\begin{aligned} \mu (t)\leq a\mathbf {E}\bigl[e^{\int _{t}^{T}\beta (s)\,\mathrm{d}s}|\mathcal {F}_{t} \bigr], \quad \mathrm{d} {\mathbf{P}}\textit{-a.s.} \end{aligned}$$

*In particular*, $\mu (t)=0$ *when* $a=0$.

### Remark 1

In order to keep the simpleness of the notations, here and here after we usually omit “d**P**-a.s.” and make random processes $\beta (\omega ,t)$ and $\mu (\omega ,t)$ abbreviated as $\beta (t)$ and $\mu (t)$ or $\beta _{t}$ and $\mu _{t}$. The notations of the other processes are similar.

### Remark 2

In the case of $\beta _{\cdot }$ being a deterministic process, i.e., $\beta _{\cdot }$ is independent of *ω*, Theorem 1 can be regarded as a direct corollary of Proposition 2. In fact, in this case, due to the compatibility of the conditional mathematical expectation (see Theorem 5.1(vii), page 81 in [11] for details) and Fubini’s theorem, for each fixed $t\in [0,T]$, it follows from (1) that

$$ \mathbf {E}\bigl[\mu (r)|\mathcal {F}_{t}\bigr]\leq a+ \int _{r}^{T}\beta (s)\mathbf {E}\bigl[\mu (s)|\mathcal {F}_{t}\bigr] {\,\mathrm{d}}s, \quad r\in [t,T]. $$

Furthermore, let $\nu (r):=\mathbf {E}[\mu (r)|\mathcal {F}_{t}]$. Then

$$ \nu (r)\leq a+ \int _{r}^{T}\beta (s)\nu (s)\,\mathrm{d}s, \quad r \in [t,T]. $$

In this way, using $[t, T]$ instead of $[0, T]$ in Proposition 2 yields

$$ \mathbf {E}\bigl[\mu (r)|\mathcal {F}_{t}\bigr]=\nu (r)\leq ae^{\int _{t}^{T}\beta (s)\,\mathrm{d}s}, \quad r \in [t,T]. $$

Finally, letting $r=t$, $t\in [0,T]$ in the above equation, we obtain

$$ \mu (t)\leq ae^{\int _{t}^{T}\beta (s)\,\mathrm{d}s}, $$

which means that the conclusion holds true.

It is necessary to point out that this proof method is no longer applicable when the stochastic process $\beta _{ \cdot }$ in Theorem 1 is not independent of *ω*, so we need to explore the new proof method.

### Iterative method

#### Proof of Theorem 1

Let $\nu (t):=a\mathbf {E}[e^{\int _{t}^{T}\beta _{s}\,\mathrm{d}s} |\mathcal {F}_{t} ]$, $t\in [0,T]$. Then $\nu (t)$ satisfies

3 $$\begin{aligned} \nu (t)=a+\mathbf {E}\biggl[ \int _{t}^{T}\beta (s)\nu (s)\,\mathrm{d}s\Big|\mathcal {F}_{t} \biggr], \quad t\in [0,T]. \end{aligned}$$

In fact, due to the compatibility of the conditional mathematical expectation, it follows that, for each $t\in [0,T]$,

$$\begin{aligned} a+\mathbf {E}\biggl[ \int _{t}^{T}\beta _{s}\nu _{s} \,\mathrm{d}s\Big|\mathcal {F}_{t} \biggr] =&a+\mathbf {E}\biggl[ \int _{t}^{T}\beta _{s} a \mathbf {E}\bigl[e^{\int _{s}^{T}\beta _{r}\,\mathrm{d}r}|\mathcal {F}_{s} \bigr]{\,\mathrm{d}}s\Big|\mathcal {F}_{t} \biggr] \\ =&a+a\mathbf {E}\biggl[\mathbf {E}\biggl[ \int _{t}^{T}\beta _{s} e^{\int _{s}^{T}\beta _{r}\,\mathrm{d}r} { \,\mathrm{d}}s\Big|\mathcal {F}_{s} \biggr]\Big|\mathcal {F}_{t} \biggr] \\ =&a+a\mathbf {E}\biggl[ \int _{t}^{T}{\beta _{s} e^{\int _{s}^{T}\beta _{r} \,\mathrm{d}r}{ \,\mathrm{d}}s}\Big|\mathcal {F}_{t} \biggr] \\ =&a+a\mathbf {E}\bigl[-e^{\int _{s}^{T}\beta _{s}\,\mathrm{d}s}\big|_{t}^{T} |{{\mathcal {F}_{t}}} \bigr] \\ =&a\mathbf {E}\bigl[e^{\int _{t}^{T}\beta _{t}\,\mathrm{d}t}|\mathcal {F}_{t} \bigr] \\ =&\nu _{t}. \end{aligned}$$

Now, let $\varphi _{t}=\mu _{t}-\nu _{t}$. Then it follows from (1) and (3) that

4 $$\begin{aligned} \varphi _{t} \leq & \biggl\{ a+\mathbf {E}\biggl[ \int _{t}^{T}\beta _{s}\mu _{s} \,\mathrm{d}s\Big|\mathcal {F}_{t} \biggr] \biggr\} - \biggl\{ a+\mathbf {E}\biggl[ \int _{t} ^{T}\beta _{s}\nu _{s}\,\mathrm{d}s\Big|\mathcal {F}_{t} \biggr] \biggr\} \\ =&\mathbf {E}\biggl[ \int _{t}^{T}\beta _{s}\varphi _{s}\,\mathrm{d}s\Big|\mathcal {F}_{t} \biggr], \quad t\in [0,T]. \end{aligned}$$

In the sequel, we will use the mathematical induction method to prove that, for each $n\geq 1$,

5 $$\begin{aligned} \varphi _{t}\leq \mathbf {E}\biggl[ \int _{t}^{T}\frac{ (\int _{t}^{u}\beta _{s}\,\mathrm{d}s )^{n}}{n!}\beta _{u} \varphi _{u}\,\mathrm{d}u\Big|\mathcal {F}_{t} \biggr], \quad t\in [0,T]. \end{aligned}$$

First, in view of (4) and the progressively measurable property of the process $\beta _{\cdot}$, exchanging the integral order of the double integral in (4), we obtain

$$\begin{aligned} \varphi _{t} \leq & \mathbf {E}\biggl[ \int _{t}^{T}\beta _{s}\varphi _{s} \,\mathrm{d}s\Big|\mathcal {F}_{t} \biggr] \\ \leq & \mathbf {E}\biggl[ \int _{t}^{T}\beta _{s}\mathbf {E}\biggl[ \int _{s}^{T}\beta _{u} \varphi _{u}\,\mathrm{d}u\Big|\mathcal {F}_{s} \biggr]{\,\mathrm{d}}s\Big|\mathcal {F}_{t} \biggr] \\ =&\mathbf {E}\biggl[ \int _{t}^{T}\beta _{s} \int _{s}^{T}\beta _{u}\varphi _{u} \,\mathrm{d}u\,\mathrm{d}s\Big|\mathcal {F}_{t} \biggr] \\ =&\mathbf {E}\biggl[ \int _{t}^{T}\beta _{u}\varphi _{u} \int _{t}^{u}\beta _{s} \,\mathrm{d}s\, \mathrm{d}u\Big|\mathcal {F}_{t} \biggr], \quad t\in [0,T]. \end{aligned}$$

Hence, (5) is true when $n=1$. Now, assume that, when $n=k$, we have

6 $$\begin{aligned} \varphi (t) \leq \mathbf {E}\biggl[ \int _{t}^{T}\frac{(\int _{t}^{u}\beta _{s} \,\mathrm{d}s)^{k}}{k!}\beta _{u} \varphi _{u}\,\mathrm{d}u\Big|\mathcal {F}_{t} \biggr], \quad t\in [0,T]. \end{aligned}$$

We will prove that, when $n=k+1$,

$$ \varphi _{t} \leq \mathbf {E}\biggl[ \int _{t}^{T}\frac{ (\int _{t}^{u}\beta _{s}\,\mathrm{d}s )^{k +1}}{(k+1)!}\beta _{u} \varphi _{u}\,\mathrm{d}u \Big|\mathcal {F}_{t} \biggr], \quad t\in [0,T]. $$

In fact, in view of (4), (6) and the progressively measurable property of the process $\beta _{\cdot}$, we can take advantage of the formula of integration by parts to obtain

$$\begin{aligned} \varphi _{t} \leq &\mathbf {E}\biggl[ \int _{t}^{T}\frac{(\int _{t}^{u}\beta _{s} \,\mathrm{d}s)^{k}}{k!}\beta _{u}\mathbf {E}\biggl[ \int _{u}^{T}\beta _{r}\varphi _{r}\,\mathrm{d}r\Big|\mathcal {F}_{u} \biggr]{\,\mathrm{d}}u\Big|\mathcal {F}_{t} \biggr] \\ =&\mathbf {E}\biggl[ \int _{t}^{T} \biggl(\frac{(\int _{t}^{u}\beta _{s}\,\mathrm{d}s)^{k}}{k!} \beta _{u} \int _{u}^{T}\beta _{r}\varphi _{r}\,\mathrm{d}r \biggr)\,\mathrm{d}u \Big|\mathcal {F}_{t} \biggr] \\ =&\mathbf {E}\biggl[ \int _{t}^{T} \biggl( \int _{u}^{T}\beta _{r}\varphi _{r} \,\mathrm{d}r \biggr)\,\mathrm{d} { \biggl[\frac{(\int _{t}^{u}\beta _{s} \,\mathrm{d}s)^{k +1}}{(k+1)!} \biggr]}\Big|\mathcal {F}_{t} \biggr] \\ =&\mathbf {E}\biggl[\frac{(\int _{t}^{u}\beta _{s}\,\mathrm{d}s)^{k +1}}{(k+1)!} \int _{u}^{T}\beta _{r}\varphi _{r}\,\mathrm{d}r\bigg|_{t}^{T}+ \int _{t} ^{T}{\frac{(\int _{t}^{u}\beta _{s}\,\mathrm{d}s)^{k +1}}{(k+1)!}\beta _{u} \varphi _{u}\,\mathrm{d}u}\Big|\mathcal {F}_{t} \biggr] \\ =&\mathbf {E}\biggl[ \int _{t}^{T}{\frac{(\int _{t}^{u}\beta _{s}\,\mathrm{d}s)^{k+1}}{(k+1)!} \beta _{u} \varphi _{u}\,\mathrm{d}u}\Big|\mathcal {F}_{t} \biggr]. \end{aligned}$$

Above all, (5) is established.

Furthermore, it is easy to verify that the right side of (5) tends to 0 when $n\rightarrow +\infty $ by the Lebesgue dominated convergence theorem together with the condition of Theorem 1, which means $\varphi (t)\leq 0$ for each $t\in [0,T]$, that is,

$$ \mu (t)\leq \nu (t)=a\mathbf {E}\bigl[e^{\int _{t}^{T}\beta (s)\, \mathrm{d}s}|\mathcal {F}_{t} \bigr], \quad t \in [0,T]. $$

Finally, in the case of $a=0$, it follows from the above proof process that, for each $n\geq 1$,

$$\begin{aligned} \mu (t) \leq & \frac{1}{n} + \mathbf {E}\biggl[ \int _{t}^{T}\beta (s)\mu (s) \,\mathrm{d}s\Big|\mathcal {F}_{t} \biggr] \\ \leq & \frac{1}{n}\mathbf {E}\bigl[e^{\int _{t}^{T}\beta (s){d}s}|\mathcal {F}_{t} \bigr], \quad t\in [0,T]. \end{aligned}$$

Therefore, let $n\rightarrow +\infty $, then $\mu (t)\equiv 0$. The proof is then complete. □

### Integral method

#### Proof of Theorem 1

By replacing *t* in (1) with *r*, multiplying both sides of (1) by $b_{r}=\beta _{r} e^{\int _{0}^{r}\beta _{s} \,\mathrm{d}s}$, and then taking the integral on $[t,T]$ and the conditional mathematical expectation with respect to $\mathcal {F}_{t}$, it follows that, for each $t\in [0,T]$,

7 $$\begin{aligned} \mathbf {E}\biggl[ \int _{t}^{T}{b_{r}\mu _{r}\, \mathrm{d}r}\Big|\mathcal {F}_{t} \biggr] \leq & a\mathbf {E}\biggl[ \int _{t}^{T}{b_{r}\,\mathrm{d}r}\Big|\mathcal {F}_{t} \biggr]+ \mathbf {E}\biggl[ \int _{t}^{T}{b_{r}}\mathbf {E}\biggl[ \int _{r}^{T}{\beta _{s}\mu _{s} \,\mathrm{d}s}\Big|\mathcal {F}_{r} \biggr]{\,\mathrm{d}}r\Big|\mathcal {F}_{t} \biggr] \\ =&a\mathbf {E}\bigl[e^{\int _{0}^{r}{\beta _{s}\,\mathrm{d}s}}\big|_{t}^{T} |\mathcal {F}_{t} \bigr]+\mathbf {E}\biggl[ \int _{t}^{T} \biggl(b_{r} \int _{r}^{T}\beta _{s}\mu _{s}\,\mathrm{d}s \biggr)\,\mathrm{d}r\Big|\mathcal {F}_{t} \biggr]. \end{aligned}$$

On the other hand, exchanging the integral order of the double integral yields

8 $$\begin{aligned} \int _{t}^{T} \biggl({b_{r}} \int _{r}^{T}\beta _{s}\mu _{s}\,\mathrm{d}s \biggr) \,\mathrm{d}r =& \int _{t}^{T} \biggl({\beta _{s}\mu _{s}} \int _{t}^{s}{b_{r} \,\mathrm{d}r} \biggr)\,\mathrm{d}s \\ =& \int _{t}^{T}{\beta _{s}\mu _{s}\cdot } e^{\int _{0}^{r}\beta _{u} \,\mathrm{d}u}\bigg|_{t}^{s}\, \mathrm{d}s \\ =& \int _{t}^{T}{b_{s}\mu _{s}\, \mathrm{d}s}-e^{\int _{0}^{t}\beta _{u} \,\mathrm{d}u} \int _{t}^{T}{\beta _{s}\mu _{s}\,\mathrm{d}s}, \quad t\in [0,T]. \end{aligned}$$

Now, substituting (8) into the right side of (7) and rewriting this formula, we have

$$ e^{\int _{0}^{t}\beta _{s}\,\mathrm{d}s}\mathbf {E}\biggl[ \int _{t}^{T}{\beta _{s}\mu _{s}\,\mathrm{d}s}\Big|\mathcal {F}_{t} \biggr] \leq a\mathbf {E}\bigl[e^{\int _{0}^{T} \beta _{s}\,\mathrm{d}s}-e^{\int _{0}^{t}{\beta _{s}\,\mathrm{d}s}}|\mathcal {F}_{t} \bigr], \quad t\in [0,T]. $$

Hence,

$$ \mathbf {E}\biggl[ \int _{t}^{T}\beta _{s}\mu _{s} \,\mathrm{d}s\Big|\mathcal {F}_{t} \biggr] \leq a\mathbf {E}\bigl[e^{\int _{t}^{T}\beta _{r}\,\mathrm{d}r}|\mathcal {F}_{t} \bigr]-a, \quad t\in [0,T]. $$

Furthermore, in view of (1), (2) follows immediately.

The proof of the case of $a=0$ is the same as in Sect. 3.1. The proof is complete. □

### Martingale representation method

#### Proof of Theorem 1

Denote $\xi =\int _{0}^{T}\beta _{s}\mu _{s} \,\mathrm{d}s$. Then $\mathbf {E}[|\xi |]<+\infty $ by the condition of Theorem 1. By the martingale representation theorem (see Theorem 2.46, page 120 in [12] for details), it follows that there exists a stochastic process $(z_{t})_{t\in [0,T]}\in \bigcap_{\beta \in (0,1)}M^{\beta }$ such that, for each $t\in [0,T]$,

$$ \mathbf {E}[\xi |\mathcal {F}_{t}]=\mathbf {E}[\xi ]+ \int _{0}^{t}{z_{s}\cdot { \,\mathrm{d}}B_{s}}. $$

Now, let

$$ \bar{\mu }_{t}:=a+\mathbf {E}\biggl[ \int _{t}^{T}{\beta _{s}\mu _{s}\,\mathrm{d}s} \Big|\mathcal {F}_{t} \biggr], \quad t\in [0,T]. $$

Then $\bar{\mu }_{t}$ is $(\mathcal {F}_{t})$-progressively measurable, $\mu _{t}\leq \bar{\mu }_{t}$, and

$$\begin{aligned} \bar{\mu }_{t} =&a+\mathbf {E}\biggl[\xi - \int _{0}^{t}\beta _{s}\mu _{s}\,\mathrm{d}s\Big|\mathcal {F}_{t}\biggr] \\ =&a+\mathbf {E}[\xi |\mathcal {F}_{t}]- \int _{0}^{t}\beta _{s}\mu _{s}\,\mathrm{d}s \\ =&a+\mathbf {E}[\xi ]+ \int _{0}^{t}{z_{s}}\cdot { \mathrm{d}}B_{s}- \int _{0} ^{t}\beta _{s}\mu _{s}\,\mathrm{d}s, \quad t\in [0,T]. \end{aligned}$$

Next, using Itô’s formula and the fact that $\mu _{t}\leq \bar{ \mu }_{t}$ yields

9 $$\begin{aligned} {\mathrm{d}} \bigl(\bar{\mu }_{t}{e^{\int _{0}^{t}{\beta _{s}\,\mathrm{d}s}}} \bigr) =&e^{\int _{0}^{t}{\beta _{s}\,\mathrm{d}s}}[-\beta _{t}\mu _{t}\,\mathrm{d}t+z _{t}\cdot {\mathrm{d}}B_{t}+\beta _{t}\bar{\mu }_{t}\,\mathrm{d}t] \\ \geq & e^{\int _{0}^{t}{\beta _{s}\,\mathrm{d}s}}z_{t}\cdot {\mathrm{d}}B _{t}. \end{aligned}$$

By the fact that the process $(\int _{0}^{t}e^{\int _{0}^{r}{\beta _{s}\,\mathrm{d}s}}z_{r}\cdot {\mathrm{d}}B_{r} ) _{t\in [0,T]}$ is $(\mathcal {F}_{t})$-martingale, note that

$$\begin{aligned} \mathbf {E}\biggl[ \int _{t}^{T}e^{\int _{0}^{r}{\beta _{s}\,\mathrm{d}s}}z_{r} \cdot {\mathrm{d}}B_{r}\Big|\mathcal {F}_{t} \biggr] =&\mathbf {E}\biggl[ \int _{0}^{T} e^{\int _{0}^{r}{\beta _{s}\,\mathrm{d}s}}z_{r}\cdot {\mathrm{d}}B_{r} \Big|\mathcal {F}_{t} \biggr]- \int _{0}^{t}e^{\int _{0}^{r}{\beta _{s}\,\mathrm{d}s}}z _{r}\cdot {\mathrm{d}}B_{r} \\ =&0, \quad t\in [0,T]. \end{aligned}$$

Integrating on $[t,T]$ and taking the conditional mathematical expectation with respect to $\mathcal {F}_{t}$ on both sides of (9) leads to, in view of $\bar{\mu }_{T}=a$,

$$ \mathbf {E}\bigl[ae^{\int _{0}^{T}{\beta _{s}\,\mathrm{d}s}}-\bar{\mu }_{t}{e^{ \int _{0}^{t}{\beta _{s}\,\mathrm{d}s}}}|\mathcal {F}_{t} \bigr]\geq 0, \quad t\in [0,T]. $$

Furthermore, since the process $\bar{\mu }_{t}{e^{\int _{0}^{t}{\beta _{s}\,\mathrm{d}s}}}$ is $\mathcal {F}_{t}$-measurable, we have $\mathbf {E}[\bar{\mu }_{t}{e^{\int _{0}^{t}{\beta _{s}\,\mathrm{d}s}}} |\mathcal {F}_{t} ]=\bar{\mu }_{t}{e^{\int _{0}^{t}{\beta _{s} \,\mathrm{d}s}}}$, and then

$$ \bar{\mu }_{t}{e^{\int _{0}^{t}{\beta _{s}\,\mathrm{d}s}}}\leq \mathbf {E}\bigl[ae ^{\int _{0}^{T}{\beta _{s}\,\mathrm{d}s}}|\mathcal {F}_{t} \bigr], \quad t\in [0,T]. $$

So

$$ \mu _{t}\leq \bar{\mu }_{t}\leq a\mathbf {E}\bigl[e^{\int _{t}^{T}{\beta _{s} \,\mathrm{d}s}}| \mathcal {F}_{t} \bigr], \quad t\in [0,T]. $$

Thus, (2) is established.

The proof of the case of $a=0$ is the same as in Sect. 3.1. The proof is completed. □

## Application

This section will introduce an application of the stochastic Gronwall’s inequality.

We are concerned with the following one-dimensional backward stochastic differential equation (BSDE for short in the remaining):

10 $$\begin{aligned} y_{t}=\xi + \int _{t}^{T}g(s,y_{s},z_{s})\, \mathrm{d}s- \int _{t}^{T}z_{s} \cdot { \mathrm{d}}B_{s}, \quad t\in [0,T], \end{aligned}$$

where $0 < T \le +\infty $ is called the terminal time, *ξ* is $\mathcal {F}_{T}$-measurable random variable called the terminal condition, the random function $g(\omega ,t,y,z):\varOmega \times [0,T]\times \mathbf {R}\times \mathbf {R}^{d}\rightarrow \mathbf {R}$ is $(\mathcal {F}_{t})$-progressively measurable for each $(y, z)$ called the generator of BSDE (9). The solution $(y_{t},z_{t})_{t \in [0,T]}$ is a pair of $(\mathcal {F}_{t})$-progressively measurable processes and the triple $(\xi , T, g)$ is called the parameters of BSDE (9). BSDE with the parameters $(\xi , T, g)$ is usually denoted by BSDE $(\xi ,T,g)$.

### Definition 1

Assume $p>1$. If $(y_{t},z_{t})_{t\in [0,T]}\in \mathcal {S}^{p}(0,T;\mathbf {R}) \times M^{p}(0,T;\mathbf {R}^{d})$ and $\mathrm{d}{\mathbf{P}}\mbox{-a.s.}$, BSDE (9) holds true, then the processes $(y_{t},z_{t})_{t\in [0,T]}$ is called an $L^{p}$ solution of BSDE (9).

Assume that the generator *g* satisfies the following assumption:

(H) There exist two stochastic processes $(u_{t})_{t\in [0,T]}$, $(v_{t})_{t\in [0,T]}$ satisfying
  $$ \int _{0}^{T} \bigl[u_{t}(\omega )+v_{t}^{2}(\omega ) \bigr]{\,\mathrm{d}}t \leq M, \quad \mathrm{d}\mathbf{P}\mbox{-a.s.}, $$
  for some constant $M>0$ and such that $\mathrm{d}\mathbf{P}\times \mathrm{d}t\mbox{-a.e.}$, for each $(y_{i},z_{i})\in \mathbf {R}\times \mathbf {R}^{d}$, $i=1,2$,
  $$ \bigl\vert g(\omega ,t,y_{1},z_{1})-g(\omega ,t,y_{2},z_{2}) \bigr\vert \leq u _{t}(\omega ) \vert y_{1}-y_{2} \vert +v_{t}(\omega ) \vert z_{1}-z_{2} \vert . $$

### Theorem 2

*Let* $p>1$, $0< T\leq +\infty $, $\xi , \xi '\in L^{p}(\varOmega ,\mathcal {F}_{T},{\mathbf{P}})$, *g* *and* $g'$ *be two generators of BSDEs*, *and let* $(y_{\cdot }, z_{\cdot })$ *and* $(y'_{\cdot }, z'_{\cdot })$ *be an* $L^{p}$ *solution to BSDE* $(\xi ,T,g)$ *and BSDE* $(\xi ',T,g')$, *respectively*. *If* $\mathrm{d}{\mathbf{P}}\textit{-a.s.}$, $\xi \leq \xi '$ *and one of the following two statements is satisfied*:

(i) *g* *satisfies* (H) *and* ${\mathbf{1}}_{y _{t}>y'_{t}}(g(t, y'_{t}, z'_{t})-g'(t, y'_{t}, z'_{t}))\leq 0$, $\mathrm{d}{\mathbf{P}}\times \mathrm{d}t\textit{-a.e.}$;
(ii) $g'$ *satisfies* (H) *and* ${\mathbf{1}} _{y_{t}>y'_{t}}(g(t, y_{t}, z_{t})-g'(t, y_{t}, z_{t}))\leq 0 $, $\mathrm{d}{\mathbf{P}}\times \mathrm{d}t\textit{-a.e.}$,

*then*, *for each* $t\in [0,T]$, $y_{t}\le y'_{t}$, $\mathrm{d}{\mathbf{P}}\textit{-a.s.}$

### Proof

We only prove the case (i). The other case can be proved in the same way. Set $\hat{y}_{\cdot }:= y_{\cdot }-y'_{\cdot }$, $\hat{z}_{\cdot }:=z_{\cdot }-z'_{\cdot }$ and $\hat{\xi }:= \xi - \xi '$. Then

$$ \hat{y}_{t} = \hat{\xi }+ \int _{t}^{T}\bigl[g(s, y_{s}, z_{s})-g'\bigl(s, y'_{s}, z'_{s}\bigr)\bigr]\,\mathrm{d}s- \int _{t}^{T}{\hat{z}_{s}\cdot { \mathrm{d}}B _{s}}, \quad t\in [0, T]. $$

Tanaka’s formula (see page 220 in [13] for details) yields, in view of $\mathrm{d}\mathbf{P}\mbox{-a.s.}$, $\hat{\xi }^{+}=0$,

$$\begin{aligned} \hat{y}_{t}^{+} =& \hat{\xi }^{+} + \int _{t}^{T}{\mathbf{1}_{\hat{y} _{s}>0}\bigl(g(s, y_{s}, z_{s})-g'\bigl(s, y'_{s}, z'_{s}\bigr)\bigr)\,\mathrm{d}s}- \int _{t}^{T}{\mathbf{1}_{\hat{y}_{s}>0} \hat{z}_{s}\cdot {\mathrm{d}}B_{s}}- \frac{1}{2} L_{t}^{0} \\ \leq & \int _{t}^{T}{\mathbf{1}_{\hat{y}_{s}>0}\bigl(g(s, y_{s}, z_{s})-g'\bigl(s, y'_{s}, z'_{s}\bigr)\bigr)\,\mathrm{d}s}- \int _{t}^{T}{\mathbf{1}_{\hat{y}_{s}>0} \hat{z}_{s}\cdot {\mathrm{d}}B_{s}}, \end{aligned}$$

where $L_{t}^{0}$ is the semimartingale local time of the process $\hat{y}_{t}$ at 0, it is an increasing process and $L_{0}^{0}=0$. Since $\mathbf{1}_{y_{t}>y'_{t}}(g(t, y'_{t}, z'_{t})-g'(t, y'_{t}, z'_{t}))$ is non-positive, we have

$$\begin{aligned}& \mathbf{1}_{\hat{y}_{s}>0}\bigl(g(s, y_{s}, z_{s})-g' \bigl(s, y'_{s}, z'_{s}\bigr) \bigr) \\& \quad =\mathbf{1}_{\hat{y}_{s}>0}\bigl(g(s, y_{s}, z_{s})-g \bigl(s, y'_{s}, z'_{s}\bigr) \bigr)+ \mathbf{1}_{\hat{y}_{s}>0}\bigl(g\bigl(s, y'_{s}, z'_{s}\bigr)-g'\bigl(s, y'_{s}, z'_{s}\bigr)\bigr) \\& \quad \le \mathbf{1}_{\hat{y}_{s}>0}\bigl(g(s, y_{s}, z_{s})-g\bigl(s, y'_{s}, z'_{s} \bigr)\bigr), \quad \,\mathrm{d}\mathbf{P}\times \mathrm{d}s\mbox{-a.e.}, \end{aligned}$$

Furthermore, using the assumption (H) for *g*, we deduce that

$$ \mathbf{1}_{\hat{y}_{s}>0}\bigl(g(s, y_{s}, z_{s})-g \bigl(s, y'_{s}, z'_{s}\bigr) \bigr) \leq u_{s}\hat{y}^{+}_{s}+ \mathbf{1}_{\hat{y}_{s}>0}v_{s} \vert \hat{z}_{s} \vert , \quad \,\mathrm{d}\mathbf{P}\times \mathrm{d}s\mbox{-a.e.} $$

Therefore, we obtain

11 $$\begin{aligned} \hat{y}_{t}^{+} \le & \int _{t}^{T} \bigl({u_{s} \hat{y}^{+}_{s}+ \mathbf{1}_{\hat{y}_{s}>0}v_{s} \vert \hat{z}_{s} \vert } \bigr) \,\mathrm{d}s- \int _{t}^{T}{\mathbf{1}_{\hat{y}_{s}>0} \hat{z}_{s}\cdot {\mathrm{d}}B_{s}} \\ =& \int _{t}^{T}{u_{s}\hat{y}^{+}_{s} \,\mathrm{d}s}- \int _{t}^{T}{\mathbf{1} _{\hat{y}_{s}>0} \hat{z}_{s}}\cdot \biggl[ {\mathrm{d}}B_{s}- \frac{v _{s}\hat{z}_{s}}{ \vert \hat{z}_{s} \vert }\mathbf{1}_{ \vert \hat{z}_{s} \vert \neq 0} \,\mathrm{d}s \biggr], \quad t\in [0,T]. \end{aligned}$$

In the sequel, let **Q** be the probability measure on $(\varOmega , \mathcal {F}_{T})$ which is equivalent to **P** and defined by

$$ \frac{\mathrm{d}\mathbf{Q}}{\mathrm{d}\mathbf{P}}:=\exp \biggl\{ \int _{0}^{T}{\frac{v_{s}\hat{z}_{s}}{ \vert \hat{z}_{s} \vert } \mathbf{1}_{ \vert \hat{z} _{s} \vert \neq 0}\cdot \,\mathrm{d}B_{s}}-\frac{1}{2} \int _{0}^{T}\mathbf{1} _{ \vert \hat{z}_{s} \vert \neq 0} \vert v_{s} \vert ^{2}\,\mathrm{d}s \biggr\} . $$

It is worth noting that ${\mathrm{d}\mathbf{Q}}/{\mathrm{d}} \mathbf{P}$ has moments of all orders since ${\int _{0}^{T}}{v^{2}}(s) \,\mathrm{d}s\leq M$, $\mathrm{d}\mathbf{P}\mbox{-a.s.}$ By Girsanov’s theorem, under **Q** the process

$$ \tilde{B}(t)=B_{t}- \int _{0}^{t}{\frac{v_{s}\hat{z}_{s}}{ \vert \hat{z}_{s} \vert } \mathbf{1}_{ \vert \hat{z}_{s} \vert \neq 0}\,\mathrm{d}s}, \quad t\in [0,T], $$

is a Brownian motion. Moreover, the process

$$ \biggl( \int _{0}^{t}{\mathbf{1}_{\hat{y}_{s}>0} \hat{z}_{s}}\cdot \mathrm{d} {\tilde{B}}s \biggr)_{t\in [0,T]} $$

is an $(\mathcal {F}_{t},\mathbf{Q})$-martingale. Thus, taking the conditional expectation with respect to $\mathcal {F}_{t}$ under **Q** in (10), we obtain, for each $t\in [0,T]$,

$$ \hat{y}_{t}^{+} \leq \mathbf {E}_{\mathbf{Q}} \biggl[ \int _{t}^{T}{u_{s}\hat{y} ^{+}_{s}\,\mathrm{d}s}\Big|\mathcal {F}_{t} \biggr], \quad { \mathrm{d}}\mathbf{Q}\mbox{-a.s.} $$

Thus, it follows from Theorem 1 that $\hat{y}_{t}^{+}=0$, d**Q**-a.s, and then $y_{t} \le y'_{t}$, $\mathrm{d}\mathbf{P}\mbox{-a.s.}$ for each $t\in [0,T]$ since **Q** is equivalent to **P**. Theorem 2 is then proved. □

### Remark 3

It follows from Theorem 2 that if *g* satisfies the assumption (H), then the $L^{p}$ solution of BSDE $(\xi ,T,g)$ must be unique.

## References

[1] Gronwall T.H. (1919). Note on the derivatives with respect to a parameter of the solutions of a system of differential equations. Ann. Math..

[2] Bellmen R. (1943). The stability of solutions of linear differential equations. Duke Math. J..

[3] LaSalle J. (1949). Uniqueness theorems and successive approximations. Ann. Math..

[4] Bihari I. (1956). A generalization of a lemma of Bellman and its application to uniqueness problems of differential equations. Acta Math. Hung..

[5] Pachpatte B.G. (1988). Inequalities for Differential and Integral Equations.

[6] Chen C.J., Cheung W.S., Zhao D. (2009). Gronwall–Bellman-type integral inequalities and applications to BVPs. J. Inequal. Appl..

[7] Lin S.Y. (2013). Generalized Gronwall inequalities and their applications to fractional differential equations. J. Inequal. Appl..

[8] Zhang Y.H., Huang J., Xu X. (2014). A generalized discrete fractional order Gronwall inequality. Math. Pract. Theory.

[9] Xu R., Zhang Y. (2015). Generalized Gronwall fractional summation inequalities and their applications. J. Inequal. Appl..

[10] Fan S.J., Jiang L., Tian D.J. (2011). One-dimensional BSDEs with finite and infinite time horizons. Stoch. Process. Appl..

[11] Kallenberg O. (1997). Foundations of Modern Probability.

[12] Pardoux E., Răṣscanu A. (2014). Stochastic Differential Equations, Backward SDEs, Partial Differential Equations.

[13] Karatzas I., Shreve S.E. (1991). Brownian Motion and Stochastic Calculus.

